# Ordinary Icons: Public Discourses and Everyday Lives in an Anxious Europe

**DOI:** 10.1111/aman.13207

**Published:** 2019-03-11

**Authors:** Anouk de Koning, Anick Vollebergh

**Affiliations:** ^1^ Department of Anthropology and Development Studies Radboud University Nijmegen The Netherlands; ^2^ Department of Anthropology and Development Studies Radboud University Nijmegen The Netherlands

## Abstract

Across Europe, ethnically diverse neighborhoods figure as key sites in racialized public debates that imagine the nation as white and nonwhite citizens as foreign to the body politic. Drawing on research in Antwerp and Amsterdam, we examine how public discourses come to shape the lives of residents in such iconic sites. We propose the notion of *ordinary iconic figures* as a way to understand these connections. Ordinary iconic figures represent generic types that populate national narratives and connect the local and the national as well as the individual and larger categories. These figures come into being in public discourses but are taken up beyond the sphere of politics and media. Such ordinary iconic figures offer commonsense frames for understanding urban landscapes, carve out speaking positions, and come to haunt residents’ sense of self as iconic shadows. They thereby help transport the inequalities laid out in public discourses into people's everyday lives. [*urban anthropology, political anthropology, racialization, iconic figures, Amsterdam, Antwerp, Europe*]

## RESUMEN

A través de Europa, vecindarios diversos figuran como sitios claves en los debates públicos racializados que imaginan la nación como blanca y los ciudadanos no blancos como ajenos al cuerpo político. Basadas en investigación en Amberes y Ámsterdam, examinamos cómo los discursos públicos llegan a moldear las vidas de los residentes en tales sitios iconizados. Proponemos la noción de *figuras icónicas ordinarias* como una forma de entender estas conexiones. Las figuras icónicas ordinarias representan tipos genéricos que pueblan las narrativas nacionales y conectan lo local y lo nacional así como lo individual y categorías más grandes. Estas figuras llegan a existir en discursos públicos, pero se toman mas allá de la esfera de la política y los medios de comunicación. Tales figuras icónicas ordinarias ofrecen marcos de sentido común para entender los paisajes urbanos, tallar posiciones para hablar, y venir a atormentar el sentido de sí mismos de los residentes como *sombras icónicas*. Ellas por lo tanto ayudan a transportar las desigualdades presentadas en discursos públicos a las vidas cotidianas de las personas. [*antropología urbana, antropología política, racialización, figuras icónicas, Ámsterdam, Amberes, Europa*]

## NEDERLANDSE SAMENVATTING

In heel Europa spelen grootstedelijke buurten met veel bewoners met een migratie‐achtergrond een sleutelrol in geracialiseerde debatten over de samenleving. Op basis van veldwerk in Antwerpen en Amsterdam analyseren we hoe publieke vertogen de levens van buurtbewoners in dergelijke iconische buurten beïnvloeden. We introduceren het concept van de ‘alledaagse iconische figuur’ als een manier om deze verbanden te begrijpen. Alledaagse iconische figuren representeren algemene types waarmee verhalen over de natie verteld worden. Ze verbinden het lokale en het nationale, het individu en de categorie. Ze ontstaan in publieke vertogen, maar duiken vervolgens ook op buiten de politiek en media. Alledaagse iconische figuren bieden mensen kaders om hun stedelijke omgeving mee te begrijpen, scheppen posities waarvandaan zij kunnen spreken en hangen als een schaduw over de wijze waarop mensen zichzelf zien. Alledaagse iconische figuren reproduceren daarmee de ongelijkheden die in publieke vertogen gecreëerd worden in de persoonlijke leefsfeer. [*stedelijke antropologie, politieke antropologie, racialisering, iconische figuren, Amsterdam, Antwerpen, Europa*]

Over the last few decades, immigration and an increase in ethnic diversity have been the focus of an anxious politics across Europe (De Koning and Modest 2017). Socioculturally and ethnoracially diverse urban landscapes and convivial subcultures (Nowicka and Vertovec 2013) contrast with resurgent forms of nationalism that imagine the nation as consisting of white natives and envision nonwhite citizens as foreign to the body politic (Silverstein 2005). Understanding this combination of exclusionary national imaginaries and growing urban diversity is one of the main challenges when studying contemporary Europe (cf. Valluvan 2016a). How do such forms of conviviality relate to the exclusionary, stigmatizing, and racialized understandings of society laid out in public discourses?

Anxious public discourses about the fate of society are often elaborated with reference to specific sites: urban neighborhoods with sizeable migrant populations. These sites provide a fertile setting for the staging of national dramas that involve a relational cast of characters that often includes young men with migrant or Muslim backgrounds, “ordinary nationals,” and left‐leaning elites. With their particular race, class, and gender markers, these characters, which we call *ordinary iconic figures*, embody the main protagonists in a battle over national belonging.

Drawing on ethnographic research in Antwerp and Amsterdam, we examine the impact of the iconization of certain ethnically diverse urban neighborhoods and ask how particular public discourses come to infuse and shape the lives of their residents. We propose the notion of ordinary iconic figures as a way to understand these connections. While there are parallels between our argument and linguistic work on the crafting of identities in the context of various language ideologies (Bucholtz 2004; Bucholtz and Hall 2005; Dick and Wirtz 2011), this article sets out to make another kind of intervention. It explores the connection between highly politicized discourses about the nation and everyday lives in an effort to understand how a particular political moment shapes senses of self, other, and one's surroundings, and how the inequalities set out in political narratives come to shape people's lives.

Below, we first introduce our ethnographic sites and their place in public discourses about changing national society. This also allows us to flesh out our conception of ordinary iconic figures. We then discuss how these figures move out of the realm of mass‐mediated public discourses into urban everyday lives. We demonstrate that ordinary iconic figures resurface in everyday situations as frames for reading and inhabiting urban life. They resurface as speaking positions and as iconic shadows that haunt people's perceptions of themselves and others.

## ICONIC SITES, NATIONAL DRAMAS

The anxious discourses about diversity and immigration that are now dominant in Europe rely on a host of highly mediatized stories about particular places, incidents, and characters (Lentin and Titley 2011). Though diversity has long ceased to be a phenomenon only of urban centers, the narrative of a nation threatened by migrants, or of native nationals impeded in their “way of life,” is often told through highly repetitive stories about particular urban neighborhoods, *banlieues*, housing estates, and so on. Media images and stories stage these urban localities as iconic sites where dramas are played out that are at once local and national. These sites come to be read as “both emblem and microcosm” of multicultural sociality (Keith and Cross 1993, 9). Projecting these national dramas onto the backdrops of actual housing estates or streets, evoking their tangible details, brings these discourses to life and makes them concrete and “real” to media publics. Iconized diverse neighborhoods convey and produce “a tangle of ambivalent feelings, sentiments and common sense ‘knowledge’” about the nature and future of the diverse nation (Keith 2005, 27). Echoing an older binary of sensibilities implicated in the trope of the city as a place of both disrupted moral order and of enlightenment and openness (Williams 1973), iconic diverse neighborhoods are marshaled for competing political projects in Europe's highly polarized political arena; they can be made to symbolize both dystopian and utopian visions of the diverse nation.

Amsterdam's Diamantbuurt, where De Koning conducted her research in 2011–2012, is such an iconic setting.^1^ The Diamantbuurt's national notoriety started with a series of 2004 news reports that focused on a clash between an anonymous group of “Moroccan youths” (or, in extreme‐right politician Geert Wilders's terminology, “Moroccan street terrorists”) and an “ordinary” (white, working‐class) Dutch couple, who were given the quintessentially Dutch pseudonyms “Bert” and “Marja.” Local and national were folded in on each other as a journalist framed the Diamantbuurt story as exemplary of claims that ordinary Dutch men and women no longer feel at home in their own country (Duyvendak 2011). The Diamantbuurt was presented as proof of the failure of multicultural society, of the arrogant wrong‐headedness of leftist elites in supporting multiculturalism, and, above all, of the trouble with “Moroccans” and the burden they present to society, or “the Dutch” (see De Koning 2013). The neighborhood's iconicity lingered long after the 2004 conflict had blown over, with new incidents being framed by—and, in turn, feeding—the neighborhood's notoriety.

The Diamantbuurt is a small, centrally located neighborhood with picturesque 1920s worker housing. Before 2004, it had never made the headlines, even though it was locally known as a gathering place for rowdy youths. The neighborhood stood out from its gentrified surroundings on multiple counts: as a bulwark of social housing, as relatively poor, and as relatively ethnically mixed (De Koning 2015). So‐called *autochtoon* residents (meaning an individual born in the Netherlands whose parents were also born in the Netherlands) made up the largest demographic slice. They were generally older, and a number were on disability benefits. A relatively large share of the neighborhood's young had Moroccan‐Dutch backgrounds, and half of them grew up in minimum income households. At the time of research, this neighborhood demography was about to change, as social housing was steadily sold off and policymakers hoped to attract much‐desired (white, highly educated) “new urbanites” to repair the neighborhood's faulty social fabric (De Koning 2015).

As the Diamantbuurt story continued to make headlines, the Diamantbuurt young men became the latest incarnation of troublesome “Moroccan youths,” a trope that has been at the center of fierce debates and deep anxieties about Dutch society. “Moroccan youths” have been routinely linked to nuisances in public space and to criminal activities. In media and public discourses, they have been portrayed as the ultimate “big‐city pests” (De Koning 2016; Pakes 2010). At least since the 1990s, “Moroccan youths” have served as proof of the failure of the multicultural Netherlands and the trouble that people from “non‐Western” migrant backgrounds pose for “the Dutch.”

The figure of the Moroccan youth was elaborated in contradistinction to that of the “ordinary” white Dutch, which, again, was developed in contrast to the left‐leaning elite. This leftist elite could easily transmute into “politicians,” “the government,” or overtly soft social workers. This is illustrated well by a 2011 cover of a mainstream weekly magazine, which, in gray tones, shows the out‐of‐focus silhouette of a hooded figure coming toward the reader, his middle finger raised. Superimposed in big letters are the words, “Lessons from the Diamantbuurt. Or: how we should deal with Moroccan punks [*etters*] and other street scum.” The cover proposes a particular subjectivity, viewpoint, and relationality, asking how “we” (the Dutch) should deal with “them” (Moroccan punks).

The Diamantbuurt illustrates how media stories stage particular places as iconic sites that give color, texture, and a sense of tangible reality to political discourses about multicultural failure. Such narratives are organized around generic types of residents. We encounter not only the figure of the national everyman with an implicit range of raced, classed, gendered, and religious features that we know from studies of populism (Laclau 2005; Mouffe 2005; Oudenampsen 2013), but also figures who reference groups that are racialized as nonnative or strangers, as well as representatives of more elite sections. These do not emerge as singular figures. They function as a cast of relational characters that together enable the telling of a story that transcends the local and comes to symbolize an unfolding *national* drama.

Oud‐Borgerhout, the Antwerp borough where Vollebergh conducted her research between 2008 and 2010,^2^ shows us a more polyvalent iconization. Oud‐Borgerhout is a large, densely populated borough within the nineteenth‐century belt around Antwerp's medieval inner city, composed mostly of terraced housing dotted with some mid‐rise postwar social housing apartment buildings, small parks, and playgrounds. Suffering greatly from urban flight in the 1970s, Oud‐Borgerhout changed from a lower‐middle‐class municipality to a deprived district of Antwerp made up of elderly white residents and Moroccan labor migrants and their families (Swyngedouw 2000). At the time of research, up to 70 percent of the population had a migrant (mostly Moroccan) background, and poverty and unemployment were high, while moderate gentrification had attracted white young families and the creative class.

Ever since the 1980s, Borgerhout has played an iconic role in the Flemish national political and media landscape, as well as in fiction and film. In contrast to the Diamantbuurt, however, it has been deployed in contrasting narratives about the future of the nation, organizing very different sensibilities about diverse neighborhood life (Beyen 2015; Vollebergh 2016a). The extreme‐right Flemish nationalist party Flemish Block (later renamed Flemish Interest) was the first to bring the trope of “Borgerokko” into the political arena. It used “Borgerokko” as a symbol of the threat of immigration, invoking two opposed but mutually constitutive ordinary iconic figures: that of the drug‐pushing and criminal migrant youth, or “stranger,” with his alien culture and language, and the “ordinary Fleming” (*gewone Vlaming*), who, on account of the former, no longer feels safe or at home in “his own” neighborhood. After the landslide victories of Flemish Block in the early 1990s, journalists started to take to Borgerhout, where Flemish Block had won 40 percent of the votes, not to explore the immigrant threat but to confront and explain the “black menace” of the rise of the extreme right itself. Flemish Block voters were framed as both problematically racist and as suffering from deprivation and social disintegration (Swyngedouw 2000; Uitermark 2014, 1426–1427). At the same time, mainstream political parties adopted aspects of Flemish Block discourse. “Borgerokko” became iconic as a somber “migrant ghetto” and deprived neighborhood in which migrant youth were pitted against underprivileged racist elderly Flemish Block voters.^3^

In the context of increasing gentrification and the success of the pro‐diversity ecological party Green!, also based in Borgerhout, a third iconic figure was introduced. The white middle‐class “positivo,” consciously choosing to move to Borgerhout, became a fixture of the Borgerhout narrative, set against the figure of the elderly, disgruntled “negativo” (Rotthier 2001). This character was at the center of a new and more optimistic plotline introduced by Green! politicians in which “Borgerhout” (no longer “Borgerokko”) symbolized precisely the richness and potential of the diverse city (Beyen 2015, 250).^4^

The Borgerhout case highlights the fact that the national staging of particular neighborhood scenes in Europe takes place in a highly charged political climate. The localized national dramas elaborated through neighborhoods like Borgerhout and the Diamantbuurt are always already told from a partisan political perspective against other, contesting perspectives. The imageries of “Borgerokko” depended on a fixed cast of three mutually constitutive characters: the elderly, angry, white working‐class resident; the “Moroccan‐Muslim youth” loitering in the street; and the optimistic, pro‐diversity, new‐middle‐class gentrifier. Though this cast was more or less fixed, the meaning of the separate characters, and the role they were made to play, shifted according to who was telling what story about Borgerhout, and to what end. The meaning of ordinary iconic figures is thus more elusive and less stable than their ubiquity and evocative tangibility suggests, reflecting the political contestation from which these figures were born.

The Diamantbuurt and Borgerhout narratives rely on the figures of the dangerous and racialized youth, the victimized ordinary national, and different versions of the leftist elites. These figures resurface in other European contexts (Ewing 2008) and beyond (Chavez 2013). There are also some significant differences. The early Far Right wins in Antwerp created the troubling figure of the Flemish Block voter, who continued to draw anxious attention alongside that of the “stranger,” and was framed as equally in need of “integration.” The Amsterdam case evidenced a more univocal narrative. The figure of the “ordinary Dutch” couple was not problematized as “racist,” even though some locals argued that “Bert and Marja” were indeed racist. In contrast to Borgerhout, where gentrification had introduced the optimistic figure of the “new Belgian,” the leftist elite in the Amsterdam case figured as a distant instigator of the multicultural trouble but not (yet) as local residents. It is telling that narratives about Amsterdam North, a city district that combines a problematized white working class with a rapid influx of predominantly white, highly educated gentrifiers (Wekker 2017), do approximate the Borgerhout narrative. Specific local demographics and contestations thus present compelling stages for certain narratives about the nation. Neighborhoods move in or out of the public eye according to their fit with the political narratives of the moment.

## ORDINARY MEN AND WOMEN AS ICONS

Iconic figures like the ones staged in Amsterdam and Antwerp can be important political techniques, primarily because they allow people to relate in very personal and affective ways to larger national narratives. As Haugbolle and Kuzmanovic (2015, 5) argue, “iconic figures form a pivotal point in the production of various kinds of mass‐mediated publics… . They spark debate, inspire deep emotions and attachment to cultural and political projects, generate collective aspirations and set people and ideas in motion.” Most discussions about iconic figures examine well‐known public figures, particularly political leaders and intellectuals, who gain iconic status in media landscapes and political movements. Some discussions also include ordinary people who become iconic because of particular experiences that set them apart, predominantly through their death (Ekal and Eldén 2015).

Our iconic figures differ markedly from these extraordinary figures. Like the figure of the “welfare queen” (Carr 2011; Fraser and Gordon 1994), ordinary iconic figures are rarely produced through the iconization of individual people—even if they can attach themselves to named individuals at certain moments—but are rather constructed as generic types. In this sense, they are reminiscent of Asif Agha's (2011, 172–73) discussion of figures of personhood, “contingent, performable behaviors that convey icons … of personhood to those for whom they function as signs.” Agha argues:

A figure of personhood once abstracted from performance as a generic symbol may be subjected to various forms of decontextualized depiction: Its existence and characteristics may be debated, discussed, considered and re‐considered. It may acquire an official name, be used as a term of address, or targeted as a market segment. It may help configure certain paradigmatic personae … [to which] actual persons may orient [themselves]. (173)

Like their extraordinary counterparts, ordinary iconic figures mediate between the particular and the abstract, but they remain tied to categories of “ordinary” residents, whom they are taken to represent. It is their ordinariness and their generic character that give our type of iconic figures a distinct role in public discourses and public and political imaginaries. These figures are imagined as hyperreal, yet they can also stand in for entire categories. They are strongly linked to a particular locality, yet they can travel beyond the iconic sites in which they were first made to emerge on the public scene. This representative function, tying together the local and the national, the particular and the generic, enables their pivotal role in narratives about the future of the diverse nation. As they come to frame news, they are continuously revitalized by new anecdotes, incidents, and narratives, reemerging in new versions of the national drama in new iconic sites.

Ordinary iconic figures are imagined through intersecting sets of characteristics, such as class, gender, age, religion, location, and race/ethnicity, as well as sexuality. They combine linguistic, sensory, and visual cues (Dick and Wirtz 2011) in connection with particular socio‐spatial scenes. The details of such “telling” cues are rarely explicated (cf. Feldman 1991, 56). These figures are dense nodes of narrative, visual, and tactile associations, and they invite deep affective engagements. In contrast to discussions of particular iconic figures—the welfare queen or the ordinary man representing “the people” that we know from discussions of populism—our approach emphasizes the relational nature of ordinary iconic figures, who together operate as a cast of characters. As a result, the ordinary iconic figures we discuss can function as vehicles for self‐identification *and* for complex, intersectional ways of “telling” others (Aretxaga 1997; Burton 1979; Feldman 1991).

Ordinary iconic figures derive part of their strength from the fact that they suggest a shared understanding and immediate sensory identification while allowing for multiple readings and evaluations (cf. Cohen 2001). Because they are produced in a context of political contention and deep polarization, ordinary iconic figures are never fully determined. The particular story lines in which they are made to function and their moral evaluation are part of Europe's battleground over questions of migration and diversity, rendering their meaning contested. However, the degree of polyvalence is not the same for each ordinary iconic figure. Few public narratives attributed a more positive meaning to the iconic figure of the “Moroccan youth.” The twin figure of the “ordinary Fleming”/“Flemish Block voter,” in contrast, allowed for multiple readings and moral evaluations.

Ordinary iconic figures are inescapable and omnipresent. They constitute a core cast of characters that resurfaces over and over and is used across the political spectrum. These figures are never neutral but are, instead, intrinsic to contestations of Europe's multicultural present and future. What does it mean for residents to live in iconized spaces? How do the iconic narratives told about their neighborhood inform and shape their navigation of neighborhood space, their relation to fellow neighborhood residents, and their sense of themselves? In the remainder, we will describe three ways in which ordinary iconic figures help us conceptualize the complex relation between public discourses and everyday life. Ordinary icons function as frames for reading neighborhood space and “telling” people, as speaking positions, and as haunting shadows that both shape senses of self and inspire counternarratives.

## READING URBAN SCENES

Ordinary iconic figures are not just techniques that help elaborate mediatized political narratives about the diverse nation; they also provide frames through which people make sense of their own lives and surroundings and carve out positions that can be taken up and lived (cf. Mepschen 2016). The cast of characters that dominated media representations of Borgerhout resonated strongly in the vernacular categories that residents used to identify themselves and others in the neighborhood. As Vollebergh joined the exchange of small talk on Borgerhout's disheveled playgrounds, participated in neighborhood feasts and daily routines, or asked about neighborhood biographies during interviews, residents from all class and ethnic backgrounds routinely described Borgerhout as populated by “Belgians” and “Moroccans,” dividing up the former into “old” and “new” or “young” Belgians. These iconic labels gloss over much of Borgerhout's demographic complexities. Nationality, or “Belgianness,” has become an ethnic marker rather than a citizenship status in the context of the diverse neighborhood (most second‐ and third‐generation “Moroccan” residents are Belgian nationals). It also links “true” national belonging and citizenship to “autochtonous cultural norms” and, more implicitly, whiteness.

These labels not only operate as “grammars of identity and alterity” (Baumann and Gingrich 2006) but also organize distinct structures of feeling that color the minute sensibilities of everyday life (Shoshan 2008, 389). When navigating their neighborhood, Borgerhout residents experienced and construed objects, places, and people around them as tangible indices of these three categories, reading them as part of teleological plots regarding the neighborhood's future. This engendered a “somatic weaving of an ethnicized urban landscape” (Shoshan 2008, 380) that allowed residents to read directionality and meaning into otherwise inchoate emotions and actions in ways that are socially shared within particular spaces and networks.

This is well illustrated by some of Vollebergh's elderly white acquaintances, who positioned themselves as “original” Borgerhoutians and “ordinary Belgians” through a racialized narrative of neighborhood demise and replacement. Elsa, a plump woman in her seventies, provides a good example. Every Friday, she did her shopping, slowly and carefully with her walker, at the Borgerhout market, after which she met up with a small group of friends in the local pub. The walk would take her past “her” beloved street, the street where she had lived for decades before the death of her husband and a series of accidents forced her to move to a nearby senior home. As Vollebergh joined her on these walks, Elsa stopped regularly to greet friends, chat with former neighbors (the “finest Moroccans” in Borgerhout, she confided), waving at youngsters she had known since their childhoods or factory coworkers of her late husband. Her movements and greetings breathed an intense “public familiarity” (Blokland and Nast 2014), yet the story she told about Borgerhout was one of alienation and loss. She pointed out all the (Belgian‐owned) shops that had disappeared, shuddered at the sight of “Moroccans” fingering the fruit at the market stalls, complained about trash on the street, and cast annoyed looks at groups of “Moroccan” women with strollers, which, to her, were clear signs of dilapidation and an encroachment of strangeness. “All these streets here, it's only strangers moving in, it's full of them, and only a few Belgians left.”

This is not Elsa's individual reading. It is a “community discourse” (Back 1996, 29) shared and circulated among longtime residents for whom spending time in neighborhood public space is an important pastime and form of sociality. Anecdotes—about youths waiting in local parks to rob “Belgian” elderly women, about veiled women with strollers not making space on the sidewalk, about “Moroccan” children claiming playgrounds as “theirs”—were shared as part of gossip and chitchat, such as during Elsa's weekly meeting with friends in the pub. In these anecdotes, mundane scenes—teenagers loitering in a park, nonwhite children playing in a playground, mothers walking and chatting side by side—became saturated with meaning, signifying how “strangers” had come to arrogantly, sometimes violently, dominate neighborhood space at the expense of “Belgians.” Through these stories, the mundane actions of those “Moroccans” or “strangers” were read as having a particular intent: to dominate neighborhood space.

Ordinary iconic figures and the “affective economies” (Ahmed 2004) they invoke function not only as semiotics of differentiation but also as building blocks of what Mikhail Bakhtin has called “chronotopes,” tying the everyday local to particular national futures. Chronotopes invoke “a particular timespace … [triggering] an ordered complex of attributions that defines the plot (what can happen and how), the actors (who can act and how), the moral or political normative universes involved in what happens, the trajectories of plot and character development, and the resulting effects” (Blommaert 2015, 111). In this case, a plotline is built that draws heavily on the Borgerokko image introduced by Flemish Interest: Borgerhout has fallen out of urban‐village bliss and is inevitably moving toward a future of ghetto demise. “Moroccans,” and especially the iconic figure of the “Moroccan youth,” are central culprits in this plot. Experiencing their neighborhood through this plot and its concomitant structure of feeling, Elsa and other self‐identified “old Belgians” positioned themselves as victimized “ordinary nationals,” as eyewitnesses to the reality of an unfolding multicultural drama.

However, because of Borgerhout's ambiguous iconization, competing chronotopes also circulated. White middle‐class residents who identified as “new Belgians” perceived Borgerhout through a wholly different plotline and a different urban sensibility. These residents would talk to Vollebergh about living together in Borgerhout as an ethical project in and of itself (Vollebergh 2016b). “Such a neighborhood needs people who are willing to look *beyond* an image, and who also want to invest in a neighborhood that has such a negative reputation,” said Yvonne, referring to other “new” families like hers. To them, “Moroccan” stores were not markers of a dominating strangeness but a normal feature of urban life and symbol of the richness and potential of urban diversity for those who can see it. In this plot, both the figure of the (presumably Flemish Block–voting) “old Belgian” and the (underprivileged) “Moroccan” were conceived of as inert actors unable to look beyond their parochial horizon, while positioning “new Belgians” as the ones capable of realizing Borgerhout's potential as a site of urban cosmopolitan conviviality.

As these opposed plotlines demonstrate, people perceive everyday life through a particular chronotope, and some aspects move into focus and become excessively visible. Persons or scenes that are read through the lens of negative iconic figures may become objects of debate and “uncivil attention” in the form of stares, insults, and other small acts indicating that they are not accepted as part of the legitimate public (Noble 2005). In contrast, scenes and people that are not reminiscent of an iconic counterpart—for instance, residents with other migration backgrounds in the Diamantbuurt, or white Belgians who are neither “new” nor “old”—may become ideologically invisible or be forcibly read through an ill‐fitting iconic lens (cf. Bucholtz 2004). Similarly, although almost everyone was aware of the affectionate familiarities between many “old Belgians” and Moroccan‐background residents, nobody took these as indications that the plot was false or in need of change. Instead, such instances, like Elsa's fondness of her “good” Moroccan‐background neighbors, were integrated into people's narratives only as individual exceptions.

## PROVIDING SPEAKING POSITIONS

Besides providing frames for experiencing and reading neighborhood life, iconic figures also come to inform everyday lives through contradictory and unstable forms of subject formation. Ordinary iconic figures interpellate people and thereby contribute to the shaping of particular mass‐mediated subjectivities. Althusser famously illustrated his notion of interpellation with the example of a police officer hailing a person who, in turning around to heed the call, becomes a subject and subject to the law. As Purvis and Hunt (1993, 483) argue, “Interpellation does more than ‘hail,’ it situates or places subjects within specific discursive contexts.” This form of subjectification is relevant with respect to our iconic figures, who interpellate specific categories of people, thereby constructing them as particular types of subjects and situating them in the discursive worlds in which our ordinary iconic figures play foundational roles.

In line with this argument, Michael Keith (2005) notes that various “governmentalities of the city” help materialize particular racialized subjects. He recounts the example of the figure of the ethnic entrepreneur that was brought to life through municipal policy in the 1980s, allowing particular people to speak from that position and claim resources and facilities, only to be abandoned when policy turned to a new subject, the street rebel.

The case of Yassine, who was born and raised in the Diamantbuurt, and at the time of research was in his early twenties, illustrates how ordinary iconic figures carve out such speaking positions. Yassine was not one of the Diamantbuurt men who had made national headlines and was given a long sentence for serious criminal offenses; he was not even a “G,” as those who adopted gangster style and posture were called. On account of a number of minor offenses in his late teens, he had been included in the “Top600,” a city‐wide list of six hundred “young habitual offenders” who were the subject of a targeted personalized approach that combined coercion with (limited) care (De Koning 2017). Yassine was something of an exception among his peers: he had higher academic ambitions than most and said he was determined to leave his earlier “mischief” behind. His case was often discussed by local street‐level professionals, who considered him part of criminogenic networks and labeled him a repeat offender but also thought he was not beyond reform.

Yassine's life was shadowed by the figure of the criminal Moroccan youth. He was adamant about his efforts to make something of his life but felt that his criminal record (by then more than three years old) and his subsequent Top600 status made this an almost impossible feat. In the years leading up to the research, security policies in the Diamantbuurt had been stepped up, resulting in intense monitoring and swift repression of the neighborhood's young Moroccan‐Dutch men. Yassine's inclusion in the Top600 resulted in additional surveillance. “It's a vicious cycle,” he told De Koning, “no beginning, no end.” At the time, his one hope was to leave the neighborhood, where his mere presence led to police citations for being seen with the wrong crowd.

Yassine's case was often invoked by street‐level professionals to discuss questions of efficacy and justice with respect to local youth security policies. Shirin, the local outreach social worker who introduced De Koning to Yassine, championed his case and tried to make other institutional partners see the young man in a more positive light. Yassine himself was quite eloquent and repeatedly sought a dialogue with and redress from local authorities, the police, and even the mayor. These efforts had also made him a protagonist in more sympathetic media reporting. In 2013, a feature‐length article in the Amsterdam newspaper, which avidly covered all criminal news connected to the Diamantbuurt, took up Yassine's case (Paul Vugts, *Parool*, May 18, 2013). The article, titled “I Don't Belong in the Top 600,” documented his struggle against what he saw as an unjust labeling as criminal.

Youth and security actors regularly invited Yassine to act as their interlocutor. He presented an enticing paradox: a likable, articulate young man who also embodied Amsterdam's key abject iconic figure (De Koning 2016). This made him a much‐desired yet also suspect presence: a living, accessible specimen of a deeply problematized “target group.” In a similar vein, the newspaper used Yassine's case to provide input for discussions about the justice of local security policies and “Moroccan problem youths.”

Yassine's resemblance to and ambivalent enactment of the iconic figure of the Moroccan youth gave him access to a range of institutional actors. These actors inevitably understood him as representing not only his own story but also the larger generic category of “Moroccans like him.” However friendly the conversation or genuine the interest, Yassine's iconic shadow would kindle their suspicions about his real intentions, of what lay beyond what a policy official once called his “socially acceptable face.” Public discourses and youth and security policies indeed created a position from which Yassine could speak and gain privileged access to important professional and political figures. But the abject iconic figure that provided that access also undercut his credibility and ensured he remained the object of insistent police surveillance and repression.

## HAUNTING SHADOWS

Yassine's case hints at the sticky (Ahmed 2004, 127) quality of ordinary iconic figures. We suggest the notion of iconic shadows (Vollebergh 2016a) as a way of getting at the complex relation between public discourses about the nation and negotiated, interactional senses of self. Building on the work of Thomas Blom Hansen (2012) and Sara Ahmed (2007), and drawing on Frantz Fanon's (1986) conceptualization of the impact of racial categories and racialized readings of the self in public life, this notion emphasizes the haunting quality of ordinary iconic figures in everyday lives. In highly mediatized neighborhoods that are made to figure as symbols of the predicament of the diverse nation, residents become caught in a “cultural economy of (imputed) gazes” (Hansen 2012, 6). Not only do they start to read others in light of the cast of characters populating the mediatized narratives and images about their neighborhood, but they also have to navigate the possibility or the sense of being read *themselves* in light of such ordinary iconic figures, whether by embodied passersby or by an imputed, abstract gaze as they navigate neighborhood space. The concept of iconic shadows refers to this sense of always potentially being perceived not as individuals or persons, but as “reducible to a phenotype, a cultural cipher, or a racialized shadow or doppelgänger” (Hansen 2012, 7).

If in the Diamantbuurt the weight of an iconic shadow was almost exclusively placed on Moroccan‐background youths, in Borgerhout, due to the more ambivalent cast of characters of its competing mediatized narratives, all categories of residents to some degree were confronted with an iconic shadow that was at once sticky and elusive. The way in which the iconic figure of the “deprived Flemish Interest voter” operated as a haunting shadow for “old Belgian” interlocutors is a case in point.

Since the rise of the neo‐nationalist and populist right in Flanders and across Europe (Gingrich and Banks 2006), the “Flemish Interest voter” and similar figures (voters for UKIP, the German NPD, Front National, or the Dutch PVV) have symbolized the threat of a racist element within the national “we” that was assumed to have been left behind. As Nitzan Shoshan (2016) argues with respect to Berlin, overlapping political and academic discourses constitute the “extreme‐right voter” as a deeply classed and raced figure associated with specific sites. Populist right‐wing support is thus ascribed to the white working class and to specific regions, symbolically absolving the nation or society of the moral stain of racism (Ceuppens 2003, 622–641; Haylett 2001; Hewitt 2005). Simultaneously, mainstream political parties have increasingly adopted “new realist” discourses (Prins 2002) that center on the iconic figure of the “ordinary” native citizen who feels his way of life is threatened by migrants. This figure is understood not as racist but as representing the “reality” of failed multiculturalism, which a politically correct elite has not dared to address (Arnaut et al. 2009). In Borgerhout's economy of gazes, where working‐class whiteness is overdetermined by the overlap of both discourses, the iconic figures of the “deprived, racist, Flemish Block voter” and that of the suffering “ordinary Fleming” were closely connected and could easily slip into one another. Moroccan‐background interlocutors told Vollebergh how, when the news about Flemish Block's electoral success in Borgerhout broke, they would walk the streets thinking, “every third Belgian wants me out.” Residents positioning themselves as “ordinary Fleming” and perceiving Borgerhout through the plot of demise simultaneously sensed that this very perception made them vulnerable to being read as a “racist Flemish Block voter,” whether by other passersby or by powerful publics beyond the neighborhood.

Take Fons and his wife, for example. Both of them had been raised in Borgerhout and were now in their early sixties. Fons took over the local family business, and they continued to live there as they raised their children. They saw themselves as respectable citizens fighting a lonesome, unwinnable battle against the ever‐encroaching indecency, filth, and danger that had turned their beloved uprightly Catholic and tight‐knit Borgerhout into “Borgerokko.” For the couple, this neighborhood change did not just mean becoming surrounded by “strangers” but also generated a deeply uncanny sense of gradually being made into a deviation themselves. “We have become the odd ones out,” Fons exclaimed, “but we used to be *normal*!” This eviction from a normative position ensued through multiple, intersecting sets of imputed gazes.

First, to have become the “odd ones out” was tied to the disconcerting sense of being read by fellow residents in ways, and along norms, that were not one's own. As “Moroccans” came to populate neighborhood space, the markers through which these “old Belgians” fashioned themselves as decent, normal, and normative neighborhood residents (their impeccably clean house, their volunteer work in the Catholic parish) seemed to lose their meaning. “Old Belgian” interlocutors were convinced that “Moroccan” fellow residents instead viewed “Belgians” or “Flemings” like themselves with a mixture of indifference and religious aversion. Ina, a retired civil servant in her sixties who had lived in Borgerhout for more than thirty years, elaborated this sense of being made to appear as insignificant and inferior, of literally not being noticed in the eyes of “Moroccan” passersby. “The new generation, the women … they are really arrogant, like, ‘[If you want to pass], you step off the sidewalk, you old woman, we won't move an inch for you’ … And if you're assertive and say, ‘I'd like to pass,’ then you're easily labeled a racist; we've already experienced that ourselves.”

What gave such moments of explicit hailing as “racist Belgians” by Moroccan fellow residents their force, however, is that they seemed buoyed by a dominant, abstract political gaze, one that was not confined to the neighborhood but was connected to national discussions and policies. This imputed dominant gaze was linked to the presence of “new Belgians” in Borgerhout and their alternative sensibilities, the “Greens” as Fons called them, referring to the wins of the Green! party in the district council. “Those with their cargo cycles, you never see them,” said Daniella, a woman in her early thirties who was born and raised in Borgerhout, as she sat with her kids in the playground outside her house. “Then it's easy to say that everything is nice and well here.” “Old Belgian” interlocutors sensed that in the contrast with the perception of “new Belgians” of Borgerhout as a *desirable* neighborhood, their own experience of Borgerhout as a deeply problematic ghetto gained a new meaning. Instead of securely signifying their self‐definition as normal, respectable residents battling neighborhood demise, it linked them to the figure of the “overly negative” disgruntled and deprived Flemish Block supporter, incapable of properly appreciating the potentials of the globalized, diverse city.

This was an elusive, haunting sense of misrecognition. At times ascribed to politics, politicians, the elite, the Antwerp City administration, or “the Left,” the source of this imputed dominant gaze was often left undefined. “It looks nice, to be leftist, it looks nice to be alternative,” said Fons, without specifying whose perception he meant, “but I'll tell you honestly: if we'd had the money, we would've been gone from here a long time ago. We're Christians, that doesn't mean we're racists!”

These “old Belgian” residents illustrate exceptionally well that the uncanniness of having an iconic shadow lies not in the experience of misrecognition, pure and simple. In the strongly overdetermined and polarized context of Borgerhout, Fons had come to think of himself as “Belgian” and drew upon the figure of the “ordinary Fleming” to claim respectability and truth. However, he was also aware that other Borgerhout residents and powerful publics beyond Borgerhout told very different stories that link this “ordinary Fleming” to the figure of the racist Flemish Interest voter. Under the weight of the imputed gazes of “strangers,” “Greens,” or some undefinable abstract gaze (“they,” “politics”), he sensed that his “Belgianness” could suddenly and uncontrollably be made to appear as something quite different, returning to him a distorted reflection of himself. Viewing himself through other people's eyes, he sensed, without ever being quite sure, that he was made to play an iconic role in stories about the diverse neighborhood and the future of the nation that were not his own.

## GROUNDS FOR CONTESTATION

Unlike the ambivalent reading of the “old Belgian” figure, the negative conception of “Moroccan youths” was dominant and hardly contested in Dutch public debates except for a few critical but marginal voices. Other valuations, such as the affective attachments and sense of community conveyed by Moroccan‐Dutch Diamantbuurt residents, or even an appreciation of exotic otherness (an important feature of “new Belgians” public discourses in Borgerhout), were barely able to gain traction in public debates.

In Amsterdam, the iconic figure of the Moroccan youth was construed through particular sartorial styles and phenotypical and embodied characteristics (big, padded, hooded coats, “light‐colored” features). It was most often imagined in the plural, as part of an anonymous band that inhabits urban street corners, terrorizes passersby, heckles women, and behaves aggressively toward gays and Jews. This plural existence was an important aspect of the figure's iconicity. It gave it a less than fully human nature and presented it as a collective threat to Dutch citizens. In contrast, its opponents, “ordinary Dutch Bert and Marja,” were individuals with proper names.

The iconic figure of the “Moroccan youth” was primarily read in racialized terms, ignoring other significant markers, such as gender, class, age, and location. As a result, the marginalized position attached to this iconic figure became a seemingly natural characteristic or a logical outcome of his imagined ethnic habitus, and the trouble projected onto this figure came to stick to the entire community or all those identified as “Moroccan.” What did it mean for residents of Moroccan descent to live with the constant shadow of the iconic figure of the Moroccan youth? How did they engage with this figure?

The iconic figure of the Moroccan youth seeped into people's understandings of themselves and their surroundings.^5^ It always haunted the stories of Moroccan‐Dutch residents, which focused both on the particular young men they knew and on the larger iconic figure that shadowed them. Their stories reflect a haunting sense of misrecognition that is reminiscent of W. E. B. Dubois's description of double‐consciousness in *The Souls of Black Folks*: “It is a peculiar sensation, this double‐consciousness, this sense of always looking at one's self through the eyes of others, of measuring one's soul by the tape of a world that looks on in amused contempt and pity” (quoted in Pittman 2016).

Take, for instance, Naoual, who was in her late teens at the time of the research. Naoual had grown up with many of the young men who were the focus of media and policy discourses. She had been a regular at the local youth center from a young age and was close to the local youth workers. Naoual was their success story: a local “Moroccan” girl who had successfully charted her own life and kept out of trouble, unlike many of the young men and several of the young women in her surroundings.

At the time of research, the long prison sentence meted out to a local young man, Majid, for, among other things, the torture of a former criminal partner, once again drew significant media attention to the neighborhood. When De Koning asked Naoual about Majid, she choked up. “The news about Halima's brother really shocked me… . How can you be so heartless to do something like that to someone?” Naoual stopped talking for a moment, allowing her feelings of distress to pass. She continued:

If someone can do that, you know that something is not right with that person. Simply as a human being … I think you can do much for money, but to go this far.… I read about the extortion, what he did with that iron.… That is heartless, isn't it? … I thought: “That Majid, who used to hang out [at the square] and tell his sisters to go home because it was getting late.”

Naoual wondered who could commit such heartless acts. He must not be right in his head, she concluded, thereby countering implications of ethnic pathology and paradoxically reclaiming a sense of humanity for Majid.

Samira, in her late twenties, had grown up in the Diamantbuurt, but upon marriage had moved away to a newer, more middle‐class area. She had fond childhood memories of the neighborhood and said she still missed it badly, even though with social housing being sold at unaffordable rates, she could not think of moving back. She wondered, looking back, how some of the young men she grew up with could have ended up going down the wrong path, while others had been able to make good careers. “One has become a lawyer. And you think, ‘Hey, that's also a friend of that guy who's been in jail.’ So I don't think something went wrong there. I also don't think it has anything to do with home. It's not the entire family that's in jail. Things did work out with other family members.” Samira questioned assumptions about the criminogenic nature of the neighborhood and its Moroccan‐Dutch community as well as ideas regarding the culpability of the family. By refuting these explanations, she contested the common naturalization of “Moroccan criminality.”

Like Naoual and Samira, many younger Moroccan‐Dutch women questioned the portrayal of Moroccan‐Dutch young men as universally troublesome and habitually deviant. They assertively disputed the stigmatization and marginalization of these local young men and pointed instead to the punitive course of the authorities, expressed in the way the police approached the neighborhood and its young men. They also drew attention to the intense human tragedy for the families and the young men themselves. Their narratives present one way to deal with the haunting iconic figure of the deviant Moroccan youth and the forms of misrecognition they witnessed. While they could hardly deny the resemblance of Majid and other young men with criminal careers to the iconic figure, they did contest the story line.

The older generation was less likely to contest the stigmatizing racialized and naturalized imagery that was constitutive of the iconic figure of the Moroccan youth. Many were instead haunted by the possibility that their son, cousin, or neighbor would reemerge as that iconic double and wondered anxiously what had gone wrong and who was to blame. While many pointed to growing hostility in Dutch society, they also questioned their own parenting. In an interview with De Koning's research assistant Hakima Aouragh, Mr. Bensalah, a well‐respected man in his seventies, explained why his children had managed to stay out of trouble:

Bensalah: Allah protected them, and I also did not let them interact with other kids in the street too much.

Aouragh: Were you very strict with them?

Bensalah: No, too strict is not good either. You have to improve [them].… We don't know how to educate children. Our blood is warm, we are easily angered. You have to deal with children in a gentler way, correct them, and not only with anger. That's why, to be honest, we can't educate them in the right way. We can't educate them like the Dutch do. The Dutch do not hit their children, we do, and that's where things go wrong.… But Alhamdulillah [thank God], I never … [hit them].

Mr. Bensalah and other first‐generation migrant parents worried intensely about the future of their children and the best ways to ensure their success and stop them from taking the wrong path. In Borgerhout, this anxiety had also enveloped second‐generation parents, who told Vollebergh of their frantic efforts to keep their sons away from “bad” peers. Yet, like Naoual and Samira, they also angrily denounced how their sons, brothers, and peers would summarily be dismissed as “bad Moroccan youths” by teachers or police officers based on mere appearances. Both in Borgerhout and in the Diamantbuurt, the haunting figure of the deviant Moroccan youth had become an enduring part of our interlocutors’ lives. Residents with Moroccan backgrounds were insistently interpellated by highly stigmatizing racialized discourses organized around the figure of the troublesome Moroccan youth. They engaged such haunting forms of misrecognition in generationally specific ways.

In contrast to the dominant negative reading of the figure of the Moroccan youth, and things “Moroccan,” more generally, Borgerhout's “old Belgians” could invoke a discourse that positioned them as “ordinary” and victims of a changing urban fabric and nation. In their case, the haunting quality of their iconic doubles lay not only in the ability to momentary fix into place but also in its ambiguity. It created a constant sense of the *possibility* of becoming visible in distorted ways in the eyes of others on the street but never being quite sure whether and how one does. In contrast, the racialized and deeply problematized iconic shadow of the Moroccan youth imposed itself in rather univocal ways, leaving our Moroccan‐background interlocutors few options other than militant contestation and deep self‐doubt.

Ordinary iconic figures thus help reproduce and materialize the inequalities elaborated in public and political discourses in intimate ways. It allows some to claim a place as valued subjects deserving of sympathy and protection, while it positions others as morally suspect and in need of surveillance.

## CONCLUSION

Across Europe, discourses about migration, diversity, and the nation manifest themselves in a paradoxical manner. Political claims to a post‐race society or to an essentially tolerant European civilization, couched in liberal and culturalized terms, turn out to provide the very ground for the current profusion of racialized exclusionary discourses and policies (Brubaker 2017; Lentin and Titley 2011; Valluvan 2016b).

Demonstrating the racialized premises of these discourses has proven insufficient to grasp how they are lived and negotiated in the context of urban life. We have proposed the concept of the ordinary iconic figure to capture how such public discourses come to inform senses of self and other in contemporary Europe.

Ordinary iconic figures help create tangible and deeply felt narratives about the future of national society by evoking concrete urban scenes with a cast of mutually constitutive characters. They connect the local and national, and the individual and collective, in insistent yet ambiguous ways that are deeply embedded in antagonistic political narratives. Across Europe, the key characters of anxious stories of multicultural failure are young men, who are racialized as nonnative and non‐European. They are counterposed to white, often working‐class “natives” who are said to suffer their presence. This cast of characters frequently also includes the figure of the cosmopolitan elite.

These ordinary iconic figures are imagined through a range of intersecting characteristics, but in today's Europe, they are often understood primarily in racialized terms, proposing racialized national narratives. In some cases, a religious or class reading may also be foregrounded, as the figures of the leftist elite and “old Belgian” show. These framings are crucial in positioning specific figures vis‐à‐vis the national community or society as “natives” or “strangers,” as exemplary actors, or as burden. However, because they are part and parcel of antagonistic narratives, the valorization of these figures may vary and is a prime expression of political contention.

While some ordinary iconic figures, such as the “ordinary Fleming” or “old Belgian,” attain different meanings and affective textures depending on the particular politicized narrative of which they are part, others, such as the figure of the “Moroccan youth,” have more unambiguous meanings. The impact of these figures, the agentive possibilities that they enable, and their haunting qualities thus differ considerably. Racialized and classed narratives of belonging and strangeness, and of differential rights to the nation and care of the state, come to inform, often in ghostly ways, residents’ senses of self and other.

The iconic neighborhoods in which we worked represent extreme cases that help elucidate the personal and intimate reproduction of the social inequalities set out in public discourses. Outside of such iconic neighborhoods, ordinary iconic figures may be less inescapable. However, the notion of ordinary iconic figures, with their varying degrees of ambiguity or fixity, may prove similarly useful in tracing connections between political discourses, everyday lives, and senses of self in other sites.

Offering building blocks for different and sometimes opposed neighborhood narratives, including people's own “community discourses” (Back 1996, 29), ordinary iconic figures provide openings for agency. They generate speaking positions that allow certain people to claim a platform, a place at the table, or a share of resources. Such speaking positions continue to be limited, however, by the narratives that produce them in the first place. Moreover, as haunting döppelgangers, ordinary iconic figures shadow residents and may fix them into place. Ordinary iconic figures, we argue, contribute to the materialization of the inequalities predicated in public discourses in everyday, intimate ways.

## NOTES

1 The research involved eighteen months of participant observation at the local youth center and in neighborhood and local council meetings, interviews with young and old residents, social workers and policy officials, and analyses of media reporting and policy documents. De Koning was especially close to a number of social workers, who helped her understand the workings of local policy networks. Hakima Aouragh, who assisted in the research and knew many residents from an earlier stint as community worker in the neighborhood, conducted a number of interviews in Moroccan.

2 The research consisted of fourteen months of fieldwork in Oud‐Borgerhout and one other Antwerp neighborhood. It involved spending time in neighborhood public space, meeting and conversing with people informally on squares and playgrounds, and participating in neighborhood initiatives, community center activities, and local policy meetings. In addition, sixty interviews were held with a range of Borgerhout residents (both longtime and relatively new, white, and of Moroccan background), street‐level professionals, and policy officials.

3 “Als het er zo goed is, ga er dan wonen: Borgerhout,” *HUMO* Nr. 2826, 3 November 1994, 36.

4 A third narrative featuring Borgerhout was proposed by several migrant based organizations and political parties, most famously the Arab European League. Using Borgerhout as a highly symbolic stage for demonstrations and political actions, the AEL introduced an alternative narrative in which Moroccan‐Muslim youths figured not as thugs or menace but as victims of racism and as rightful claimers of the rights and belonging denied to them by Flemish society (Jacobs 2005). Despite significant media traction, this narrative did not take hold because AEL leader Abu Jahjah was criminalized and images of AEL demonstrations were framed as riots by irrationally violent youths.

5 An early, longer version of this argument can be found in De Koning (2016).

## References

[aman13207-bib-0001] Agha, Asif . 2011 “Large and Small Scale Forms of Personhood.” Language & Communication 31 (3): 171–80.

[aman13207-bib-0002] Ahmed, Sara . 2004 “Affective Economies.” Social Text 22 (2): 117–39.

[aman13207-bib-0003] Ahmed, Sara . 2007 “A Phenomenology of Whiteness.” Feminist Theory 8 (2): 149–68.

[aman13207-bib-0004] Aretxaga, Begona . 1997 Shattering Silence: Women, Nationalism and Political Subjectivity in Northern Ireland. Princeton, NJ: Princeton University Press.

[aman13207-bib-0005] Arnaut, Karel , Sarah Bracke , Bambi Ceuppens , Sarah De Mul , Nadia Fadil , and Meryem Kanmaz . 2009 Een leeuw in een kooi. De grenzen van het multiculturele Vlaanderen [A lion in a cage: The limits of multicultural Flanders]. Antwerpen: Meulenhoff.

[aman13207-bib-0006] Back, Les . 1996 New Ethnicities and Urban Culture: Racisms and Multiculture in Young Lives. London: UCL Press Limited.

[aman13207-bib-0007] Baumann Gerd, and Gingrich André, eds. 2006 Grammars of Identity/Alterity: A Structural Approach. London: Berghahn Books.

[aman13207-bib-0009] Beyen, Marnix . 2015 “Greetings from Borgerokko. An Antwerp Neighborhood as a National Icon of Globalization and Anti‐globalism” In Local Memories in a Nationalizing and Globalizing World, edited by Beyen Marnix and Deseure Brecht, 244–257. London: Palgrave Macmillan.

[aman13207-bib-0010] Blokland, Talja , and Julia Nast . 2014 “Belonging in Berlin's Mixed Neighbourhoods.” International Journal of Urban and Regional Research 38: 1142–59.

[aman13207-bib-0011] Blommaert, Jan . 2015 “Chronotopes, Scales, and Complexity in the Study of Language in Society.” Annual Review of Anthropology 44:105–16.

[aman13207-bib-0012] Brubaker, Rogers . 2017 “Between Nationalism and Civilizationism: The European Populist Moment in Comparative Perspective.” Ethnic and Racial Studies 40 (8): 1191–226.

[aman13207-bib-0013] Bucholtz, Mary . 2004 “Styles and Stereotypes.” Pragmatics 14 (2–3): 127–47.

[aman13207-bib-0014] Bucholtz, Mary , and Kira Hall . 2005 “Identity and Interaction: A Sociocultural Linguistic Approach.” Discourse Studies 7 (4–5): 585–614.

[aman13207-bib-0015] Burton, Frank . 1979 “Ideological Social Relations in Northern Ireland.” The British Journal of Sociology 30 (1): 61–80.

[aman13207-bib-0016] Carr, E. Summerson . 2011 Scripting Addiction: The Politics of Therapeutic Talk and American Sobriety. Princeton, NJ: Princeton University Press.

[aman13207-bib-0017] Ceuppens, Bambi . 2003 Congo made in Flanders? Koloniale Vlaamse visies op “blank” en “zwart” in Belgisch Congo [Congo made in Flanders? Colonial Flemish visions on “white” and “black” in Belgian Congo]. Gent: Academia Press.

[aman13207-bib-0018] Chavez, Leo . 2013 The Latino Threat: Constructing Immigrants, Citizens, and the Nation. Stanford, CA: Stanford University Press.

[aman13207-bib-0019] Cohen, Anthony P. 2001 Symbolic Construction of Community. New York: Routledge.

[aman13207-bib-0020] De Koning, Anouk . 2013 “Creating an Exceptional Problem Neighbourhood: Media, Policy, and Amsterdam's ‘Notorious’ Diamantbuurt.” Etnofoor 25 (2): 13–30.

[aman13207-bib-0021] De Koning, Anouk . 2015 “‘This Neighbourhood Deserves an Espresso Bar Too’: Neoliberalism, Racialization, and Urban Policy.” Antipode 47 (5): 1203–23.

[aman13207-bib-0022] De Koning, Anouk . 2016 “Tracing Anxious Politics in Amsterdam.” Patterns of Prejudice 50 (2): 109–28.

[aman13207-bib-0023] De Koning, Anouk . 2017 “‘Handled with Care’: Diffuse Policing and the Production of Inequality in Amsterdam.” Ethnography 18 (4): 535–55. 30443198 10.1177/1466138117696107 PMC6195306

[aman13207-bib-0024] De Koning, Anouk , and Wayne Modest . 2017 “Anxious Politics in Postcolonial Europe.” American Anthropologist 119 (3): 524–26.

[aman13207-bib-0025] Dick, Hilary Parsons , and Kristina Wirtz . 2011 “Racializing Discourses.” Journal of Linguistic Anthropology 21 (S1): 2–10.

[aman13207-bib-0026] Duyvendak, Jan Willem . 2011 The Politics of Home: Belonging and Nostalgia in Western Europe and the United States. Basingstoke: Palgrave Macmillan.

[aman13207-bib-0027] Ekal, Berna , and Åsa Eldén . 2015 “From Icons to Emblematic Cases.” Middle East Journal of Culture and Communication 8 (1): 124–45.

[aman13207-bib-0028] Ewing, Katherine Pratt . 2008 Stolen Honor: Stigmatizing Muslim Men in Berlin. Stanford, CA: Stanford University Press.

[aman13207-bib-0029] Fanon, Frantz . 1986 Black Skin, White Masks. London: Pluto Press.

[aman13207-bib-0030] Feldman, Alan . 1991 Formations of Violence: The Narrative of the Body and Political Terror in Northern Ireland. Chicago: University of Chicago Press.

[aman13207-bib-0031] Fraser, Nancy , and Linda Gordon . 1994 “A Genealogy of Dependency: Tracing a Keyword of the U.S. Welfare State.” Signs 19 (2): 309–36.

[aman13207-bib-0032] Gingrich, André , and Marcus Banks . 2006 Neo‐Nationalism in Europe and Beyond: Perspectives from Social Anthropology. New York: Berghahn Books.

[aman13207-bib-0033] Hansen, Thomas Blom . 2012 Melancholia of Freedom: Social Life in an Indian Township in South Africa. Princeton, NJ: Princeton University Press.

[aman13207-bib-0034] Haugbolle, Sune , and Daniella Kuzmanovic . 2015 “Introduction.” Middle East Journal of Culture and Communication 8 (1): 5–11.

[aman13207-bib-0035] Haylett, Chris . 2001 “Illegitimate Subjects? Abject Whites, Neoliberal Modernisation, and Middle‐Class Multiculturalism.” Environment and Planning D: Society and Space 19 (3): 351–70.

[aman13207-bib-0036] Hewitt, Roger . 2005 White Backlash and the Politics of Multiculturalism. Cambridge: Cambridge University Press.

[aman13207-bib-0037] Jacobs, Dirk . 2005 “Arab European League (AEL): The Rapid Rise of a Radical Immigrant Movement.” Journal of Muslim Minority Affairs 25 (1): 97–115.

[aman13207-bib-0038] Keith, Michael . 2005 After the Cosmopolitan? Multicultural Cities and the Future of Racism. New York: Routledge.

[aman13207-bib-0039] Keith, Michael , and Malcolm Cross . 1993 “Racism and the Postmodern City” In Racism, the City and the State, edited by Cross Malcolm and Keith Michael, 1–30. London: Routledge.

[aman13207-bib-0040] Laclau, Ernesto . 2005 “Populism: What's in a Name?” In Populism and the Mirror of Democracy, edited by Panizza Francisco, 33–49. London: Verso.

[aman13207-bib-0041] Lentin, Alana , and Gavan Titley . 2011 The Crises of Multiculturalism: Racism in a Neoliberal Age. London: Zed Books.

[aman13207-bib-0042] Mepschen, Paul . 2016 “The Culturalization of Everyday Life: Autochthony in Amsterdam New West” In The Culturalization of Citizenship, edited by Willem Duyvendak Jan, Geschiere Peter, and Tonkens Evelien, 73–96. London: Palgrave Macmillan.

[aman13207-bib-0043] Mouffe, Chantal . 2005 “The ‘End of Politics’ and the Challenge of Right‐Wing Populism” In Populism and the Mirror of Democracy, edited by Panizza Francisco, 50–71. London: Verso.

[aman13207-bib-0044] Noble, Greg . 2005 “The Discomfort of Strangers: Racism, Incivility and Ontological Security in a Relaxed and Comfortable Nation.” Journal of Intercultural Studies 26 (1–2): 107–20.

[aman13207-bib-0045] Nowicka, Magdalena , and Steven Vertovec . 2013 “Comparing Convivialities: Dreams and Realities of Living‐With‐Difference.” European Journal of Cultural Studies 17 (4): 341–56.

[aman13207-bib-0046] Oudenampsen, Merijn . 2013 “Explaining the Swing to the Right: The Dutch Debate on the Rise of Right‐Wing Populism” In Right‐Wing Populism in Europe: Politics and Discourse, edited by Wodak Ruth, KhosraviNik Majid, and Mral Brigitte, 191–207. London: Bloomsbury.

[aman13207-bib-0047] Pakes, Francis . 2010 “Global Forces and Local Effects in Youth Justice: The Case of Moroccan Youngsters in Netherlands.” International Journal of Law, Crime and Justice 38 (3): 109–19.

[aman13207-bib-0048] Pittman, John P. 2016 “Double Consciousness.” Stanford Encyclopedia of Philosophy website. https://plato.stanford.edu/entries/double-consciousness/.

[aman13207-bib-0049] Prins, Baukje . 2002 “The Nerve to Break Taboos: New Realism in the Dutch Discourse on Multiculturalism.” Journal of International Migration and Integration 3 (3–4): 363–79.

[aman13207-bib-0050] Purvis, Trevor , and Alan Hunt . 1993 “Discourse, Ideology, Discourse, Ideology, Discourse, Ideology …” British Journal of Sociology 44 (3): 473.

[aman13207-bib-0051] Rotthier, Rudi . 2001 Hotel Fabiola. Een verslag uit Borgerhout [Hotel Fabiola: A report from Borgerhout]. Amsterdam/Antwerpen: Atlas.

[aman13207-bib-0052] Shoshan, Nitzan . 2008 “Placing the Extremes: Cityscape, Ethnic ‘Others’ and Young Right Extremists in East Berlin.” Journal of Contemporary European Studies 16 (3): 377–91.

[aman13207-bib-0053] Shoshan, Nitzan . 2016 The Management of Hate: Nation, Affect, and the Governance of Right‐Wing Extremism in Germany. Princeton, NJ: Princeton University Press.

[aman13207-bib-0054] Silverstein, Paul A. 2005 “Immigrant Racialization and the New Savage Slot: Race, Migration, and Immigration in the New Europe.” Annual Review of Anthropology 34:363–84.

[aman13207-bib-0055] Swyngedouw, Marc . 2000 “Belgium: Explaining the Relationship between Vlaams Blok and the City of Antwerp” In The Politics of the Extreme Right: From the Margins to the Mainstream, edited by Hainsworth Paul, 121–43. London: Pinter.

[aman13207-bib-0056] Uitermark, Justus . 2014 “Integration and Control: The Governing of Urban Marginality in Western Europe.” International Journal of Urban and Regional Research 38 (4): 1418–36.

[aman13207-bib-0057] Valluvan, Sivamohan . 2016a “Conviviality and Multiculture: A Post‐Integration Sociology of Multi‐Ethnic Interaction.” Young 24 (3): 204–21.

[aman13207-bib-0058] Valluvan, Sivamohan . 2016b “What Is ‘Post‐Race’ and What Does It Reveal about Contemporary Racisms?” Ethnic and Racial Studies 39 (13): 2241–51.

[aman13207-bib-0059] Vollebergh, Anick . 2016a “Strange Neighbors: Politics of ‘Living Together’ in Antwerp.” PhD dissertation, University of Amsterdam.

[aman13207-bib-0060] Vollebergh, Anick . 2016b “The Other Neighbour Paradox: Fantasies and Frustrations of ‘Living Together’ in Antwerp.” Patterns of Prejudice 50 (2): 129–49.

[aman13207-bib-0061] Wekker, Fenneke . 2017 “‘We Have to Teach Them Diversity’: On Demographic Transformations and Lived Reality in an Amsterdam Working‐Class Neighbourhood.” Ethnic and Racial Studies 2 (1): 89–104.

[aman13207-bib-0062] Williams, Raymond . 1973 The Country and the City. Oxford: Oxford University Press.

